# Extremely Severe ME/CFS—A Personal Account

**DOI:** 10.3390/healthcare9050504

**Published:** 2021-04-27

**Authors:** Whitney Dafoe

**Affiliations:** Independent Researcher, 433 Kingsley Ave., Palo Alto, CA 94301, USA; whitney@whitneydafoe.com

**Keywords:** ME/CFS, extremely severe ME/CFS, severe ME/CFS, myalgic encephalomyelitis, chronic fatigue syndrome, personal account

## Abstract

A personal account from an Extremely Severe Bedridden ME/CFS patient about the experience of living with extremely severe ME/CFS. Illness progression, medical history, description of various aspects of extremely severe ME/CFS and various essays on specific experiences are included.

## 1. Biography

**Figure 1:**
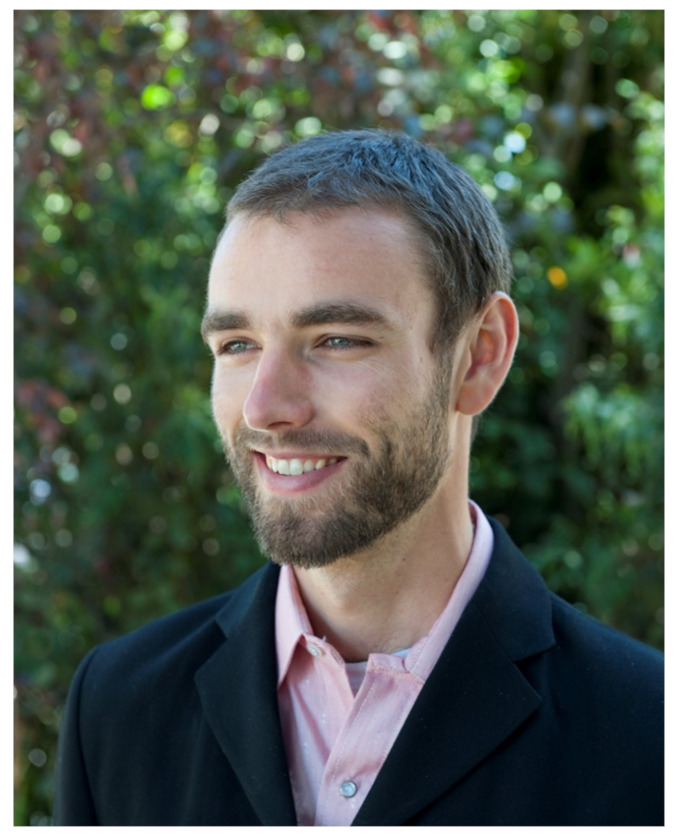
Whitney Dafoe Before Severe ME/CFS.

Whitney Dafoe (see Figure 1) studied photography at Bennington College and The San Francisco Art Institute. His award-winning work in photography and film has been published and exhibited worldwide. Whitney first got ME/CFS at age 21, which made his education and photographic pursuits much more challenging and his ultimate goal of being a war and documentary photographer impossible. His condition worsened in 2009, going from mild to moderate ME/CFS. It then quickly deteriorated into severe ME/CFS in 2012. In 2014, it worsened again into extremely severe ME/CFS. In April of 2020, Whitney saw an improvement, back to severe ME/CFS, from the drug Abilify and, although still bedridden, is able to write for a limited time most days.

## 2. Introduction to ME/CFS

**Figure 2:**
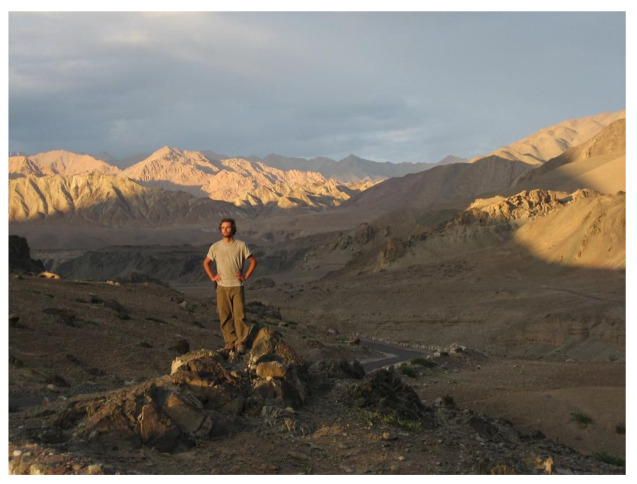
Himalayas.

I have been struggling with health problems since 2004, when I was 21. Every time I traveled, my health seemed to plummet. However, I have always been inspired and dedicated and never thought I’d wind up where I am now. Therefore, I kept going, kept pushing myself to do everything I wanted to do. A trip to India in 2006 (See Figure 2) made the illness much worse. From 2009–2012, ME/CFS progressed to a moderate state. I started a wedding photography business in 2009 when I realized I could no longer hold a full-time job, thinking that it was a blessing in disguise because, once I got my health back, I would be making money doing something I loved. After a year, things were looking really good business wise, but it took me longer and longer to recover from the intense physical requirements of shooting a wedding. In 2010, when I couldn’t recover in a week in order to shoot the next wedding, I decided I had to give it up and move back in with my parents, both heartbreaking decisions because of what they represented. For the next 2 years, I was bedridden much of the time, with my health and mobility slowly decreasing. In 2012 I was forced to rest in bed most of the day, saving up energy for little bits of projects, or working on some photographs for a half hour, or an hour on a good day.

After seeing countless doctors and specialists in every area of medicine I could find for 8 years, since I was 21, having blood drawn over and over again and literally hundreds of tests done, I was finally diagnosed with Chronic Fatigue Syndrome/Myalgic Encephalomyelitis by Dr. Andy Kogelnik at the Open Medicine Institute in Mountain View CA. As you know, there is no cure.

The Symptoms of Chronic Fatigue Syndrome (CFS), or Myalgic Encephalomyelitis (ME), vary from patient to patient [1]. The most fundamental symptom is debilitating fatigue that worsens after physical or mental exertion. However, fatigue is much too mild a word. I like to compare the state I was in in 2012 to staying up for two nights in a row while fasting, then getting drunk. The state you would be in on the third day—hung over, not having slept or eaten in 3 days—is close, but still better than many ME/CFS patients feel every day. “Total body shut down” would be a better phrase, because you are at a point where your body physically does not have the energy to keep going.

## 3. From Moderate to Severe

ME/CFS began for me in 2004, when I was 21 [2]. I was in a mild state for 5 years, with my main symptom being lightheadedness that worsened after cardiovascular exercise or came back after periods of remission after cardiovascular exercise.

While in India, I experienced a strange cold that never really took hold, but remained at about 20% for 2 weeks. This had never happened to me before. About 4 months later, one night I suddenly started feeling queasy and nauseous and had mild diarrhea once. This was followed by immediate exhaustion. I suddenly, overnight, developed severe ME/CFS. I slowly recovered from the symptoms to about 60% health, and then would get mild diarrhea (once) again and it would come back immediately, in full swing, and I would be in bed, unable to eat anything but liquified white rice soup.

After battling this for about 3 months, I finally wound up with pneumonia in a hospital in Calcutta, India and decided I had to come home. Upon arriving home, I immediately started getting better, and the ups and downs of getting worse-then-better stopped. I slowly recovered to about 80% of my former health. I still could not do cardiovascular exercise, or I would risk the lightheadedness coming back.

I then learned of a doctor from a close friend of mine, who he claimed was a sort of miracle worker and had often cured undiagnosed illnesses in people before. In hopes of regaining my full health, I flew to Guatemala to see this Doctor. After being there for about a month, the same thing that happened in India happened again. After a meal, I got mild diarrhea, my stomach shut down, and I lost all energy. I stayed there in this condition for about a week, barely hanging on, until I decided to return home. Again, upon returning home, I immediately got better. However, I did not return to the same state I was in after returning from India. I was a bit worse. I was weaker and my stomach was less functional.

In 2009, while working for Environment California, fundraising on the streets, there was an extremely cold spell (for the Bay Area). Every other worker wound up getting a cold. I didn’t. However, sometime after that, the same symptoms of the strange 20% cold I got in India came back and my health quickly started slipping back into the state I was in in India and Guatemala, though I did not get as severely ill. My health stopped deteriorating when it was about half as severe as the worst I was in India and Guatemala. At this point, I had moderate ME/CFS symptoms. I was mostly housebound but could walk a short distance to get food and do grocery shopping, cook for myself and take care of myself, although I had to spend large portions of the day resting in my room. This state continued for about 2.5 years, slowly getting worse until it was so difficult to take care of myself that I decided to move in with my parents, hoping that if I lightened my required work load and stayed under my energy limits, my condition might improve. It actually did, a little bit (see Appendix A.2).

Then, I took Rituxan (Rituximab) and this wound up permanently changing the way the illness worked in my body [3,4]. ME/CFS patients who survive have to develop acute awareness of their own bodies to monitor their energy limits and how food and various stimul affect them. Before Rituxan, if I went over my energy limits, I experienced a crash that made me exhausted for the rest of the day or multiple days, but I slowly recovered from it, close to, but not quite equal to, where I was before (as I remained slightly worse).

After Rituxan, when I went over my energy limits, I experienced a much more extreme crash. Instead of a steep curve down and slowly back up, it was like going off a cliff and I did not recover: my symptoms permanently worsened. The crash was a downward line that then just leveled off and did not curve back up. It is very difficult to never go over your energy limits, especially when every time you go over them, you get permanently worse and have to relearn your new limit, which often requires going over it once to find where it is. For me, at that point, it meant getting permanently worse, so I very quickly got much, much worse, I developed pain in the muscles in the back of my legs when standing for short periods of time or walking short distances, then I lost my ability to speak, then I could only text a few words and had to use an app with pre-programmed text messages to ask for food so I only had to touch the phone a few times to send a text. I later taught my parents a routine for my food that I stuck to so that I did not have to ask for a specific thing. My diet was an ongoing, constant rotation through the same meals at the same times. This saved me from having to text.

I continued to get worse. For many months, I walked out into the yard, laid down on a lawn chair and listened to music with headphones for several hours before walking back into my room. This, and 2–3 twenty-foot trips to the kitchen, were the most I could walk per day. I later got a wheelchair, so I didn’t have to walk to get to the kitchen (see Appendix A.8).

I continued to get worse.

One night, something traumatic happened that led to me texting more than I was capable of, due to the emotions evoked by the event. This was the end of being able to walk outside or use the wheelchair to get into the kitchen. After this one event that put me over my energy limit, I was bedridden and have been ever since (see Appendix A.5).

## 4. From Severe to Extremely Severe

In my Severe state, I was bedridden and became sensitive to human contact. I could not tolerate people being in my room for more than short periods of time [5]. This got worse, and soon trips into my room to bring me food and basic necessities became too much. Before I could get my caretakers to successfully limit their trips in and out of my room, they came in and out too many times *once* and I crashed and got worse [6]. This was on Christmas Eve. I remember lying in bed on Christmas Day, not knowing how I was going to get help or food because I couldn’t tolerate people coming in my room at all anymore (the crash made me permanently worse). I just laid in bed, kind of panicked, trying to think of a solution. I eventually came up with the idea of wearing headphones playing music while they came in. This worked! It eventually morphed into earmuffs with earphones (small earbuds) inside playing white noise very softly, the combination of which did a great job blocking outside noise. From that moment on, I didn’t have anyone in my room without earphones and earmuffs. Only when I discovered Ativan and Abilify [7] in the last few months of 2020 and improved was I able to be around people without earmuffs and actually listen to them speak, but only with Ativan or when my body naturally releases adrenaline to enable me to get up to have a bowel movement in the bathroom (a 6-foot walk). I will explain this natural adrenaline release later in this article.

I continued to slowly get worse, mostly because of the fact that it was impossible to never exceed my very low energy limits. The world is not completely predictable. Sometimes, I would think for days about how to communicate something I needed or something my caretakers were (unintentionally) doing that was hurting me in a way that they would understand. I would try to think of every possible way they could interpret the signals I planned to lay out for them, and every possible reaction they could have. Then, I would try it and they would often react in the one way I hadn’t thought of, and I would have to have them come in over and over, trying to communicate what I needed in different ways. Each trip into my room hurt me and made me worse. I often used paper towels folded into arrow shapes pointing at things, but there was a lot of room for interpretation. It was extremely stressful and devastating to try so hard to stay below my limits and then have these unexpected, uncontrollable things happen that forced me beyond my limits, when I knew I would be getting worse.

### My Stomach Functionality Declining 

In 2011, my stomach was in a steady condition; in fact, it was slowly getting better after my trip to Guatemala and continued to get a bit better when I moved in with my parents, although it was still at maybe 60% of my healthy stomach functionality. Then, it suddenly collapsed and got much worse. I think this happened because of a combination of the illness getting worse and because of one single dietary change I made. In order to sleep, I had to eat right before bed, and it had to contain protein. For years, I ate yogurt right before going to sleep and it worked great. However, many ME/CFS patients talked about dairy intolerance and that was the one thing I had never cut out of my diet to see if it made me feel better. Out of desperation, I tried eating turkey patties before bed instead of yogurt. About 3 weeks later, I woke up one morning and my stomach still felt full from the turkey patties I ate the night before. This was the beginning of my stomach no longer functioning. [8,9,10,11]

After this, I slowly could eat less and less, despite being very, very careful about never eating too much and eating the things that were easiest to digest. Interestingly, my stomach wound up with a very similar pattern to my energy limits. It’s as if my stomach had PEM. My stomach had reduced capacity and reduced digesting ability. If I ever ate too much, it would be incredibly uncomfortable, and afterward my stomach’s functional limit would go down permanently, so I had to be incredibly careful. I would sometimes take 1/4 sized bites when I got towards feeling full, because one bite too much could be devastating.

It was a horrible, horrible experience, slowly being able to eat less and less. I was slowly starving. It got to the point where I could only eat yogurt and apple juice and I drenched the yogurt in maple syrup for extra calories. I discovered my stomach worked better while asleep, so I extended my sleeping time and would wake up every hour or two and eat another yogurt cup with maple syrup. Then, I’d go back to sleep. At best, doing this, I could eat about 3 cups per day. Still not enough calories. Or nutrients. But it got worse, down to one cup split between sleep. Then, no yogurt at all. I could only handle amino acid pills which I opened and dumped in my mouth. Just a few capsules filled me up. A healthy person would feel nothing from such a tiny bit of amino acid. I also took tiny sips of straight maple syrup for sugar, which helped my brain continue to function. Then, it continued to get worse, and I could only take a few tiny sips of maple syrup spaced throughout the day, just to give my brain a little fuel.

**Figure 3:**
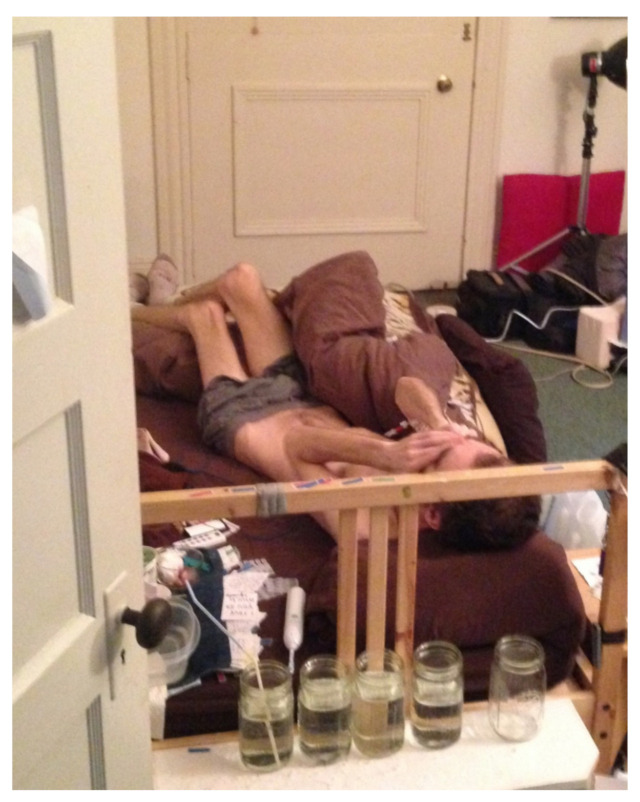
Extreme Distress and Weight Loss.

**Figure 4:**
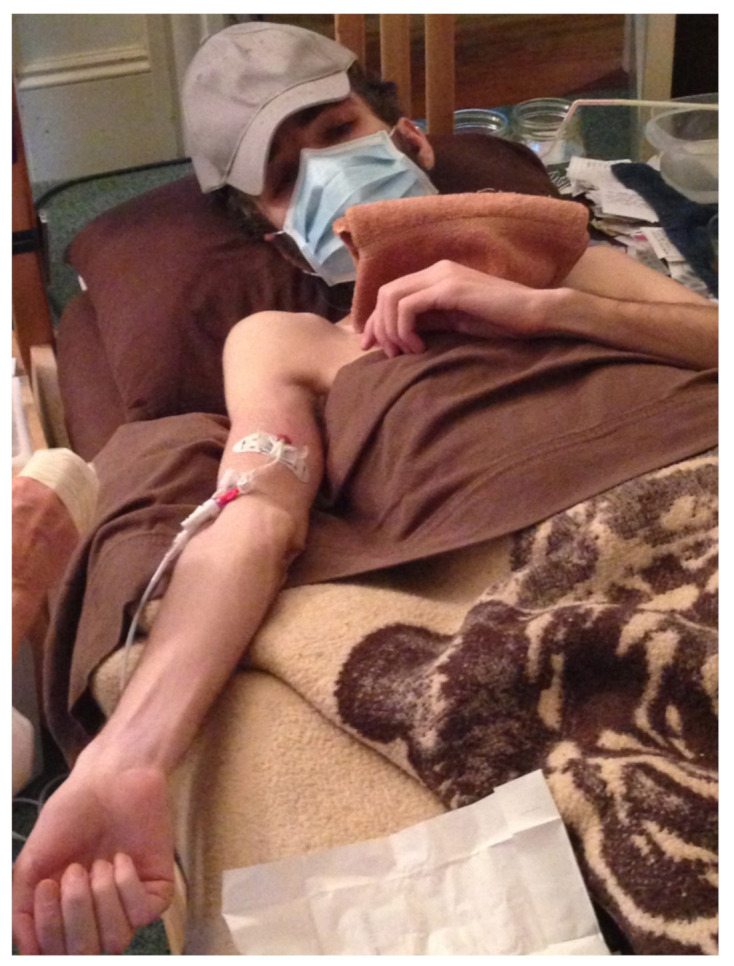
PICC Line.

Then, nothing at all. I was extremely weak and lost a huge amount of weight. I weighed 115 lbs (the same as after India) (see Figure 3). I remember being desperate but not being able to communicate. At this point, one way I was able to communicate was by using small index cards with pre-written phrases on them—generic phrases that I could use for anything, like “more/less” “please put it here”, and some specific ones as well. I was dizzy and extremely weak from starvation, and all I managed to do was put out a pre-written card on my pillow that said “Nd Hlp”. My mother, Janet Dafoe, found an in home PICC line service and they came and installed a PICC line with IV nutrition just before things started to really fall apart [12] (see Figure 4). From that point on, I have not been able to eat even a tiny crumb of food or drink a drop of water.

## 5. Having Extremely Severe ME/CFS

I fell from Severe to Extremely Severe because I passed my energy limits one day too many in a row. I had gone just over my limit multiple days in a row trying to figure out new tools and new routines to help my stomach, which was still getting worse and more uncomfortable. I felt strongly that I needed to have at least a week of calm days, but the next day, the film crew for Jen Brea’s film “Unrest” was scheduled to come. I pushed myself to let them film me despite what my body was telling me I needed. This day was one too many, and afterwards I started going downhill fast, with no bottom in sight.

When I finally leveled out, I could no longer write cards to communicate, or put out pre-written cards. I couldn’t communicate in any way. The only thing I figured out I was able to do was fold a paper towel into an arrow shape to point to something, and this only worked because a paper towel was not a tool for communication; I was re-purposing it. All communication tools were too much for me to handle.

These years are very difficult to describe in detail for multiple reasons. When I became housebound, I, at one point, realized that my thoughts were rather negative, and I realized that if I could put a negative tint on everything, I could put a positive tint on everything too. I began practicing and training my mind to think more positively. It was not easy and took practice, but this eventually became integrated into how I saw and thought about things. It was crucial for what I wound up going through (see Appendix A.4).

When I was at my worst in this two-year period, from the filming of “Unrest” until I discovered Ativan in January of 2016, I tried not to think about how bad things were; I really kept my mind focused. Thinking about my reality was extremely distressing and didn’t help anything, because it was out of my control.

This was also my least conscious period of time in the illness. I think, in time, science will show that ME/CFS patients are in a kind of hibernation state and are literally less alive. I’m confident there was less activity in my brain during this period and still is to this day compared to my healthy brain [13].

The brain is complex. When you lose a part of it, do you know it’s missing? In a way you do, but, similar to state-dependent memory, when my brain was so dysfunctional I don’t think I fully realized it.

I also don’t remember that time very well because it was so traumatizing that I blocked a lot of it out and just pushed forward, so there are multiple factors at play making that part of my illness a bit hazy.

Before describing this period of my illness, I should explain that, at this point—post Rituxan—I had developed a new kind of crash. A mental crash. When most patients refer to crashing, they are talking about what I call a body crash. A *body crash* is mental and physical exhaustion and worsening of all or most symptoms after going over one’s energy limits, followed by a gentle slope back up, but usually not back all the way to where the patient was before: the crash makes the patient permanently worse. A *mental crash* is very different. It can happen from thinking too much, from too much stimulation like noise or light. What was happening is that I got so severe that my energy limit extended into my brain. Anything that caused me to think more than my mental limit permitted caused a mental crash. It got so severe that certain subjects were too much for me to think about, and I had to try to control what my mind thought about. You know the saying that goes “Don’t think of a pink elephant”. It is very difficult not to think about something, but I had to learn to. I was in a nightmarish situation where my mind started playing tricks on me, flashing subjects I could not tolerate thinking about into my mind at the worst times and causing mental crashes. I was completely lost in a corner of my mind trying to keep my brain activity to a minimum. It was horrific.

The symptoms of this type of mental crash were usually a hot flush starting in the back of my head and moving down through my whole body, followed by an adrenaline release that temporarily made me a little better, but was later followed by my mind getting much worse. After a mental crash, I could not think at all. I was stuck in a thoughtless, feelingless void that you couldn’t imagine without experiencing it. It’s like being alive but dead at the same time. Alive only to bear witness to the absence of life in your mind and body. This would last for the entire rest of the day. One crash and I lost the only thing I had left—daydreaming of other things, other places, and creative ideas.

Because of the effect crashing had on my life, I had to put a tremendous effort into keeping to my routine as best as possible so I wouldn’t overdo it and crash and get worse. As I said, during this time, my brain was extremely sensitive to crashing from the tiniest extra interaction with caregivers or even thinking about the wrong thing, or from thinking about something for too long. I put all my focus on being perfect and then, if nothing went wrong at night when my caregivers were gone for a long period of time, all night, I could think a little bit. I remember after they left for the night, I had a little adrenaline to get fixed on my pillow and get my blankets comfortable and then it would very quickly wear off and I had to hold still. It was often a battle just to get into a position I could stay in comfortably before the adrenaline wore off, and sometimes I crashed just adjusting my pillow too much. Sometimes, I would force myself to stop before getting into a comfortable position, and then I would wind up in sometimes significant pain from this, but would try to ignore it because if I moved even one muscle, I would crash and wouldn’t be able to think. If I pulled it off, and didn’t crash, it was the best part of the day. I let my mind wander. I usually thought about making things. I have a whole business plan for multiple restaurants, buying and fixing this local natural food store, and lots more. I also thought about art projects in depth, of course. I lived for that time of daydreaming at night and somehow made a sort of life out of it.

It’s also important to note that I hadn’t been sick for nearly as long then as I have now. I had lots of hope for a recovery in the near future. I thought my father, Ronald Davis, PhD, Professor of Biochemistry and of Genetics and Director of the Stanford Genome Technology Center and now the ME/CFS Collaborative Research Center at Stanford University, would figure it out quickly. It turns out this illness is more complicated than I imagined at the time, but that hope helped carry me onward (see Appendix A.7).

During the day, it was also very difficult for me to move other than unconscious movements like adjusting in bed or scratching an itch. If I thought about any movement too much, it became extremely difficult to do because anything intentional was difficult or impossible. I had to come up with ways of “tricking” my mind into releasing adrenaline to allow me to do things like pick up the electric shiatsu massager I used on my stomach to help with the symptoms of my severe gastroparesis. I broke the movement into steps. I used various methods over time. One was to visualize the movement I was going to make over and over until suddenly my mind released the necessary adrenaline and I could tell that I could do it safely, and then I could pick it up with no problem but had to follow my pre-visualized movement. Then, I did the same for putting it on my stomach and the same for pushing the on button, then moving it to a different spot on my stomach. There were actually more steps than this in order for me to move enough to massage my stomach. It took painstakingly long hours to accomplish simple tasks.

I also became extremely sensitive to, mostly visual but also some audio, stimuli [14]. I couldn’t tolerate bright colors and had to remove everything with bright colors from my room. Everything needed to be neutral colors like white, black, brown or shades of gray. My caregivers had to wear all plain black clothing because I couldn’t tolerate any colors or patterns on them. I also became sensitive to text like logos or labels on things because it is impossible not to read text that you see; it is something we do instinctually at this age. Reading required more mental energy than I had and caused a mental crash. Due to crashing from the text I could read in my room, I wound up becoming sensitive to text I couldn’t read as well. Just knowing it was there was extremely stressful. My caregivers had to slowly and very painstakingly (often with direction from me trying to tell them where the text was, which always hurt me terribly) cover all text with black electrical tape. It remains to this day. I was also sensitive to certain sounds, especially the human voice, and during one period, any noise at all.

When I say that I became extremely sensitive to stimulation, or when you read this about severe ME/CFS, it’s not always sensitivity to the stimuli itself. The stimuli, whether it is a sound, a sight, smell, or touch, could connect my mind to something and it was this connection that often pushed my mind over its limit. The sound of people talking, for example, was too much human connection for me to tolerate. Interestingly, it was much easier to tolerate hearing people I didn’t know, like neighbors, talking. This is because it caused much less thought, because I didn’t know the people. When someone I knew spoke, their whole personality and my memories of them, etc., were forced on my mind and this was much more thought-provoking then an unknown voice.

**Figure 5:**
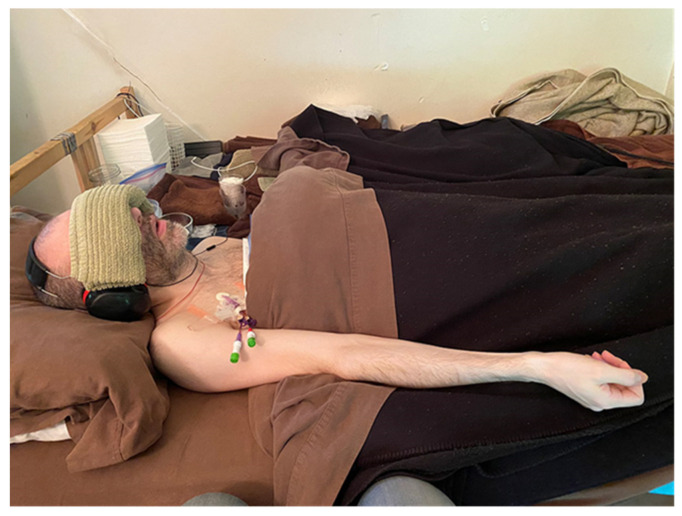
Isolating Myself from Caretaker Presence.

Sounds or other stimuli that had no mental link to anything could also be too much, simply because they are something for the brain to process. This is why I wear earmuffs and earphones playing white noise, along with a folded towel over my eyes, when someone comes into my room (see Figure 5). I need to isolate myself from the human presence and, in general, I need to isolate myself from the world. This is also why you see severe ME/CFS patients wear eye masks, baseball hats and other apparel or devices to help isolate themselves.

I also suffered from something I call “crash memory”. If I crash or get hurt from something, my mind gets what I think is a form of physically induced PTSD caused by my stress or fight/flight response being turned up as high as they could go. When I crashed from something, I developed a stress response to it and became sensitized to it, so I had to be very careful not to crash from the few things that I was able to do or think about. These “crash memories” slowly built up over time. One was getting really sensitive to noise and doing anything at the same time as hearing noise. I couldn’t turn on my stomach massager while various noises were happening. The heater air noise, a train or car going by, the click of my in-room heater turning on. I had to wait a certain amount of time after any noise before I could turn on the massager and, if I ignored it, I would crash. I slowly built up more and more sensitivity to noises and it took me forever to massage my stomach or anything else because I had to do so much waiting for gaps in the noises. If I just “did it anyways”, it would really hurt my brain and I would be in the worst brain fog of all, which created stress and compounded the whole thing. The Klonapin and Ativan I later took helped me reset these Crash Memories, so they didn’t build up. I’m now able to crash from something and let it go and do it again (with the same energy limitations as before, but no added stress or limitations).

When an ME/CFS patient becomes so severe that they are no longer able to communicate, they often start displaying what appears to be emotions like anger or rage [15]. This is a very unfortunate misunderstanding that needs to be clarified for doctors. When I lost the ability to communicate in any way, my caregivers didn’t somehow develop telepathic powers. They became out of sync and out of touch with my condition and what was happening in my life. They didn’t know when they did something that hurt me, and I had no way to tell them so they would stop doing it or do it in a different way. I was forced to resort to doing things that would connect what they did to a bad experience for them so they would stop doing it, not because they thought it was bad for me, but because they knew what would happen if they did it again. This was unfortunate, but it was the only way to survive. Babies are in a similar situation, where they have needs that don’t get met because they can’t communicate. They do similar things to what I wound up having to do. I often had to display anger and throw things that would break or otherwise make a mess that was tiring for my caregivers to clean up. I most often would dump a jar of water that was kept by my bed onto the floor, which they then had to soak up so mold wouldn’t grow. They sadly thought, at the time, that the illness was making me emotionally unstable and angry. However, I was never actually angry and always felt terrible about forcing them to clean things up, but it was the only thing I could do to change their behavior so that they stopped hurting me. This is important for doctors and caregivers to know, for two reasons—so that caregivers do not take this behavior personally and so that patients are not improperly diagnosed with mental illness by doctors. It is no more a mental illness than a baby’s cry for help (see Appendix A.6).

One thing I’ve thought about is that, despite my caregivers’ entirely good intentions and tremendous effort, my actual experience during this period was one of rather extreme abuse. It’s still true, though to a lesser extent. I got worse almost entirely because of interactions with my caregivers. This isn’t because of anything intentional on their part, but due to my sensitivity to human interaction. If I could have somehow gotten what I needed without people ever coming in my room, I would never have become so severe. I must emphasize that this was despite their good intentions, effort and sacrifices, which I have always acknowledged and been grateful for.

I don’t think I ever worried that my brain was permanently damaged. I’m not sure why. I’ve always been very in touch with my body and most of my conceptual intuitions about the illness have been proven correct by Ronald Davis (molecularly). I do still worry that brain crashes (as opposed to body crashes from overexertion, which last longer and are more of a gentle curve, not a cliff) cause brain damage, but I think the brain is resilient and can rewire itself. I try not to think about it.

My personal theory of a mental crash is as follows. When an ME/CFS patient gets severe enough, the energy limit invades the brain because use of the brain starts exceeding the energy limit. When the brain exceeds this limit, it runs out of oxygen or some other vital element, and the body responds by inducing an emergency release of adrenaline (this is the hot flush I experience) and this adrenaline increases my heart rate, which pumps more blood to my brain to avoid sudden brain death. I don’t know what this essential element is, but I feel fairly confident this is an accurate laymen’s description of what is happening. It’s an automatic emergency brain-saving reaction.

### Severe ME/CFS List in Brief—Summarizing My Quality of Life

- I haven’t left my room for 7 years, except when I have to go to the hospital to change my J-tube feeding tube out of medical necessity. I am only able to do this without dying by being sedated with Ativan the entire time, as well as Fentanyl and Versed during the procedure;
- I haven’t been touched by another human being without it hurting me in 7 years;
- I haven’t been able to speak for 7 years. I haven’t had a conversation with another human being in 8 years;
- I haven’t eaten a crumb of food or felt a drop of water in my mouth in 6 years. I’m alive because of nutrients being pumped into my body with machines and tubes;
- I haven’t taken a shower in 7 years. I clean the most necessary parts of myself with baby wipes every day and it absolutely exhausts me. I can’t handle having someone else clean me;
- I haven’t cut my own toenails in 7 years;
- I haven’t been able to hold or even touch my camera in 7 years (photography is my passion and my life);
- I haven’t peed standing up in 9 years. I haven’t walked to the bathroom to pee in 7 years. I pee in a urinal in bed;
- I haven’t made love to a woman in 9 years. I haven’t been sexual in any way in 5 years;
- I haven’t brushed my teeth in 6 years. It hurts my stomach, making it worse and putting my ability to tolerate the feeding tube at risk, which puts my life at risk;
- I haven’t seen a dentist in 9 years;
- I haven’t been able to tolerate the sound of another person’s voice without being sedated in 7 years. I wear heavy-duty earmuffs whenever my caregivers are in my room for the bare minimum of time. They can’t talk and have to be as quiet and gentle as possible;
- I haven’t felt like a human being in 7 years. All humanity has been taken from me by ME/CFS. I live only to continue living. There is no love, joy, passion or creation, only endless, numbered days; (See Appendix A.1)
- I fight to survive for all those living and dying in silence and darkness (see Appendix A.3).

## 6. Slight Improvement—Ativan and Abilify

Discovering Ativan saved me from living on the brink. After taking it for the first time, it had some sort of reset effect on my system, and all my symptoms improved permanently in addition to the temporary benefit of the drug. I had been on Total Parenteral Nutrition (TPN) through a PICC line for 1.5 years, and this is the maximum time a person can be on this type of nutrition. Some things just can’t be given through your veins. It bypasses the entire GI system, and risks liver damage. I should have been put on Total Enteral Tube Nutrition with a jejunostomy tube [16], instead of TPN and a PICC line, from the start, but my family, my doctors and I were all scared of what a trip to the hospital would do to me and we didn’t realize how much Ativan would help me. I would probably be much healthier today if I had a J tube installed then, because I would have discovered Ativan sooner, and TPN through a PICC line in your veins causes the GI tract to deteriorate and healthy bacteria to die off.

**Figure 6:**
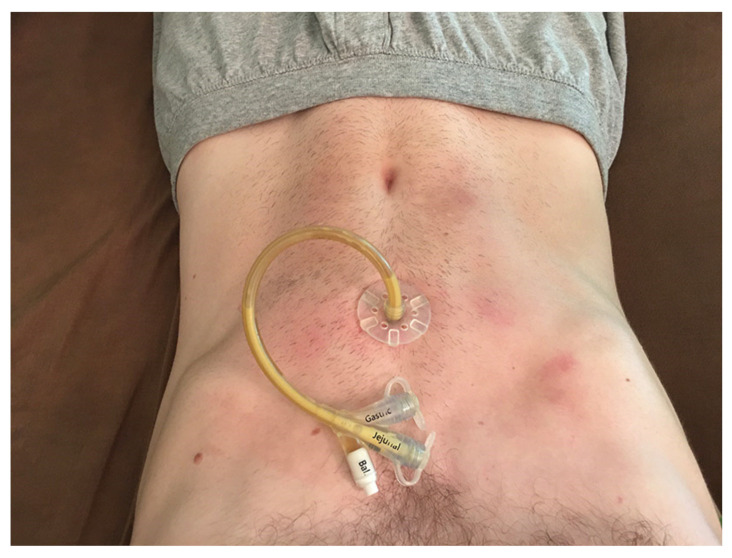
J Tube.

I took Ativan for the first time to try to make my trip to the hospital to have the J tube inserted tolerable, or at least less harmful (see Figure 6). It wound up being a game-changer for me. In addition to somehow resetting my system and permanently improving all of my symptoms, I now had a way to periodically communicate (Ativan can’t be taken all the time or you habituate to it, so I took it once every week or two). This meant that I no longer had to figure out how to communicate problems or new needs that arose; I just had to hold out and tolerate them until I could take Ativan.

Ativan mainly reduced my sensitivity so that I was able to tolerate being in the presence of people. I still could not speak and certainly could not get out of bed or do anything extra physically, but I figured out that I could gesture to communicate. This was painfully slow and took an enormous amount of energy. In time, I learned that I could also do a limited amount of writing out words in the air with my finger or onto my blanket (I still could not write on paper). I used the combination of gesturing for most things and filling in gaps that I couldn’t successfully gesture by writing them in the air. This was still hard on me, though, so I would often reduce the number of letters I had to draw out by playing a sort of “hang man”. I would write a few essential letters of a word and draw blank lines with my finger in between and try to use gesturing to help people figure out the word by guessing the other letters. Or I would do the same with a sentence, with the blank being a word I wasn’t able to get across. It was a relief to be able to finally communicate directly to people, but also traumatic in how difficult and often imprecise it was. It frequently made me feel pretty desperate.

Going to the hospital, especially for the first time, was incredible. I had no idea Ativan was going to have such a profound effect on me. I was preparing to get way worse and have a terrible time and crash horribly. Instead, I improved and was calm and got to enjoy things like seeing the sky for the first time in 6 years: all the sights of the real world out the window of the ambulance, all the healthy people working at the hospital leading healthy (or at least much healthier) lives with careers and loved ones and goals and things they were looking forward to, etc., and, a few times, seeing women my age and feeling attracted to them, and more. It was all amazing and continues to be, though it’s also exhausting and a big disruption to my routine, so it’s a mixed bag, especially coming back home and seeing the door to the outside world shut behind me. This is very difficult emotionally. I also let a lot more of myself out while on Ativan because I’m able to, and when it wears off, I have to pull it back in again and suppress myself again. It usually takes a couple days of emotional turmoil to adjust.

In the fall of 2019, I started taking Abilify at a low dose. It did nothing for the first few months because I was adding multiple medications and supplements, so I tapered up very slowly, much slower than most people when they take it now. I think I spent 6 months going from 0.25 mg to 2 mg (February 2020). After being at 2 mg for about a month, I started noticing an effect. It wasn’t the same as Ativan. I didn’t suddenly feel it like Ativan, which had an instantaneous, noticeable, drug-like effect. Abilify seemed to be changing something at a deeper level. I had more energy and could slowly tolerate more things that used to cause me stress. For the last 6–7 months, I have continued to improve, tolerating more and more things that used to make me crash from stress and over-stimulation. I can’t get out of the bed, but I can move around in bed much more than before. I can even work on some hobbies in bed on most days for some time. When I take Ativan now, I can actually listen to people talk to me, so instead of pantomiming both directions, I can listen and then pantomime back. They can say what they think I mean, and I can nod if they’ve got it. This makes it much faster but still painfully imprecise and slow for me to communicate anything to them.

Soon after my stomach completely shut down and was unable to tolerate even a drop of water, I discovered that ice helped it function better. When I started getting Total Enteral Formula with the J tube, I kept ice on my stomach for basically all of my waking hours. After being on Abilify for 6 months, I discovered through an act of brave experimentation that I could tolerate the food pump with no ice. This was a huge breakthrough, because it allowed me to move much more in bed and avoid the constant replacement of two-gallon-sized ziplock bags of ice on my entire stomach from ribs to waistline. I’m now able to get up on my knees in bed for a moment to move or reach things. I haven’t tried standing up in bed.

For my entire time with severe ME/CFS, I’ve gotten a natural adrenaline boost when I have a bowel movement that improves my condition, and, with the exception of a 6-month period when I used a commode, gives me enough energy to get out of bed and walk the 6 feet to the bathroom to go in there on the real toilet [17]. Which is, of course, a good thing for my sense of humanity and autonomy, and it’s just easier for everyone. Since Abilify has started to improve my heath, I’ve been able to harness this adrenaline to communicate after having a BM in the bathroom. I can’t interact with people for a long time, but long enough to communicate some basic things. It’s been enough that I haven’t needed to take Ativan anymore because I just wait until my next BM to communicate new needs or problems. I’ve recently added washing my feet, privates, head and face in the shower to having a BM. After the BM, I stand in the shower for a very short period and wash my feet and privates. Then, I lie down on the ground with my head sticking into the shower and my caregiver washes my head and I quickly wash my face. I don’t totally understand how my body produces adrenaline to go to the bathroom or why it isn’t able to for other things like communication or cleaning myself, but it has something to do with hardwired necessity. There is something hardwired to having a bowel movement that must push the body to get up and move somewhere else. I believe it is something we evolved to have, to help early humans move away from their sleeping place to go to the bathroom because this improved sanitation and reduced illness [18].

## 7. Important Notes

A doctor recently asked me to describe why I am unable to talk and the process behind that. The answer to this question ties into a core process of ME/CFS that is important for the world to understand because of its significance and because of how much suffering it causes patients. I haven’t spoken in 8 years, but I could talk right now if I chose to. The keyword here is *choose*. ME/CFS is not generally defined by inability, but by consequences. Everything is about Post-Exertional Malaise, mild or severe [19]. I could get out of bed and walk out the door and run right now if I chose to. I’m capable of it. The question is not what I am capable of, the question is what will happen to me afterwards (or in severe cases, might leak into the very act because it would take very little running before the reaction of severe PEM started and I might still be running when it hit, causing me to collapse or possibly die, but not from the immediate consequences of the action, but PEM). ME/CFS patients very quickly learn that their actions have consequences that occur after the fact. Patients have to learn to read and listen to their bodies.

I have learned to pre-visualize an action before doing it. When I pre-visualize performing the action, I can feel what the consequences of performing that action will be, and whether it will hurt me or not. I have incorporated this pre-visualization into every single action, big and small, and it is now how I function without having to consciously think about it. I don’t speak because, when I do this split-second pre-visualization I feel that it will hurt me.

There are some things I don’t use entirely this technique for, though. Some things feel like they might be OK, but I rely on my prior memories of doing them and what the consequences were to steer me in the right direction. One time, in 2012, when I first became bedridden, I got up out of bed to move something that had fallen over. It wound up causing severe PEM that left me exhausted and brain-fogged for days. Right now, when I pre-visualize getting out of bed and walking a few steps to get something, it feels like I maybe could, but I am scared to do it because of what happened in 2012. I can hear psychologists everywhere screaming “deconditioning!” That’s not what is happening here. It is simply intelligent learning. When I am able to get out of bed and walk a few steps to pick up something, it will be obvious to me. Getting better is a slow process with ME/CFS because of how careful patients need to be about overdoing it. When getting better, the energy limit is suddenly in unknown territory. Patients must very slowly do new things only when they feel very safe doing so. It is wiser to get better staying a bit under your absolute limits than to try to do as much as possible, and wind up making a mistake, going over the limit and then getting worse and ruining a possible upward spiral toward better health.

### 7.1. Routines

This brings me to another important part of living with ME/CFS—developing a routine. It is difficult, especially for people new to ME/CFS, to pre-visualize every little thing. It’s likely that I am better at this than other people because I am good at spatial thinking—I am a visual artist. It is difficult to always know when you will go over your limits by performing a certain action. Patients soon learn what hurts them and what is OK, and to make life easier, instead of trying to figure these limits out every hour of every day, which leads to making mistakes, patients develop routines. These routines are sets of actions a patient learns that, if performed in a certain way or order, can be done without going over their energy limits. Most people who survive ME/CFS develop routines and stick to them so that they are much less likely to go over their limits. It is a way of living that leads to much better health than constantly guessing or taking chances. It, of course, causes suffering as well because it takes spontaneity out of life. The worse a patient gets, the more every day becomes a chore of endlessly going through the same actions in the same order and in the same way, but if patients don’t develop a routine, they get worse, and lose the ability to do things they were once able to do. Do not make the mistake of diagnosing this as OCD behavior. It is a choice that ME/CFS patients make. The choice is obvious to ME/CFS patients: it is preferable to sacrifice spontaneity in order to be healthier and more active, and think more clearly.

### 7.2. The Great Beyond

Having Severe ME/CFS is so close to being dead. There’s really no other way to describe the experience I have had. I don’t think it’s something that people who haven’t had severe ME/CFS can likely understand. Looking back at who I was when I had mild and moderate ME/CFS, I’m not sure it’s something that even patients who haven’t been in the extremely severe state can fully understand. I was literally barely alive, and I am confident that, in a short time, science will prove that severe ME/CFS patients are barely alive and that ME/CFS patients, in general, are less alive mentally and physically than healthy people.

I think the only time a healthy person maybe experiences anything like this is shortly before actually dying. In that case, the person is generally in this state for a much shorter period of time and so remains much more connected to who they were, and their former lives. This is the state in which healthy people let go of their former lives and accept death, which is probably one of the reasons that suicide is so common for ME/CFS patients.

When I was severely ill, I lost so much of myself. I was holding on to fragmented memories left imprinted in my mind of who I was, but that person, in reality, didn’t exist anymore. The thought patterns and emotions and worldviews that created the person I was no longer existed. However, I was still technically alive, just enough to be conscious and bear witness to this state of non-existence.

The suffering this causes is so profound. I can only liken it to one of the hell realms described in Tibetan Buddhism. A world full of nothing but pain, loss, agony and constant never-ending challenges in holding on to what little I had left. Every mistake took me deeper into the void of nothingness.

As you know, I have recently gained back some of my mind and body. It feels like coming back from the dead. I’m in a strange state now, where bits and pieces of Whitney have come back to life but most of me has not. I’m not able to get out of bed, eat or drink water or go out and feel the world again—feel that feeling that is being alive.

I have, so far, just been riding this wave of improvement and the new-found abilities I have, like being able to write and have some semblance of connection with the world again.

However, the honeymoon phase for these improvements wore off, I started realizing how far I actually am from being Whitney again. I’ve realized that I don’t really know who I am anymore. I know who I used to be, but is that who I am? I guess I’ve realized that it is not.

The experience of being on death’s door for never-ending years has changed me permanently. I’m still not well enough to come anywhere close to fully inhabiting my own mind and body again. I don’t really know who I am. I’m in a sort of limbo right now, stripped of the person I once was and would have become, but not able to take the experiences I’ve had and create a new person out of them. I’m still a ghost, suddenly no longer fully transparent, yet, at the same time, unable to actually exist in physical form.

It’s so confusing.

While my new capabilities have improved my quality of life a small amount, I realize how much I’m still suffering and how much is still missing from my being a human being again. I’ve been so focused on my small improvements that I’ve somewhat lost touch with how far away the world still is. When I think about it now, it’s hard for me to even imagine what it would be like to be fully healthy again, out in the world again, alive again.

I don’t know who I am going to become. One thing I do know is how much the experience of losing everything has taught me. I think ME/CFS is the greatest teacher I’ve ever had. I have hope that when better treatments, and then a cure, are found, I will be a much more conscious, wiser, more realized being. That person waiting to be reborn is an incredible person, and I can’t wait to see that person and be that person and contribute to the world with my whole being (see Appendix A.9).

I think this is one of the most tragic things about the high rate of suicide among ME/CFS patients. These are people who have been through something completely unique to the rest of society and have a truly unique and profound perspective to offer the human race. When an ME/CFS patient kills themselves, so much is lost from the world.

We have seen the other side. We need to stay alive so that we can join the world again and share what is really out there in the great beyond with the rest of humanity. We have an incredible understanding of what life is. How precious and fleeting it is, how little time we have, and more. These are lessons that most people never learn, and we need to teach the rest of humanity how sacred the life they have truly is.

## Appendix A. Articles I Have Written about ME/CFS

### Appendix A.1. What Patients Often Go Through

- Loss of some or all family;
- Loss of all friends;
- Loss of job;
- Loss of hobbies;
- Loss of loved ones/relationships;
- Loss of things that used to define who you were;
- Loss of connection to the world;
- Loss of sense of dignity;
- Loss of ability to do anything physical (this includes chores, sports, outdoor activities, using your legs as transportation, and in more severe patients—self care like showers, keeping clean in general, brushing teeth, changing clothes, changing socks, etc.);
- Loss of ability to think and remember the way you used to, your mind lost from you in what is often called a “fog”;
- Accordingly, loss of your personality;
- Loss of your sense of self and sense of humanity;
- Prejudice from everyone in a patient’s life, accusations from everyone in a patient’s life of the illness being “in their head”, even after decades of illness;
- A complete lack of support from society. There is no safety net for ME/CFS patients because most patients aren’t diagnosed and even when they are, it is not considered a valid diagnosis [20]. If patients aren’t lucky enough to have friends/family to take care of them, they are left on their own. Even patients who do have people in their lives who are willing to make the incredible sacrifices required to take care of them, very few are prepared or trained, or the right kind of person for that job, which is incredibly difficult. Most Severe ME/CFS patients probably die or commit suicide when there literally is no hope [21]. A great number of them lose hope before this point;
- Lack of funding for research that would give patients something to hope for [22]. All of the above plus no research puts a huge burden of literal hopelessness on patients. Their condition is likely never to get better [23]; their only hope is a cure or treatments, but there’s no funding for scientists to do research to find treatments or a cure. The Open Medicine Foundation-funded research, spearheaded by Ronald Davis out of Stanford University, is the first extensive, collaborative research effort into ME/CFS [24], but it is pretty new. For the last 40 years (the illness has likely been around for much, much longer than that, but was even more covered up, prejudiced against and misunderstood, to the point that there was not even recognition of its existence), there has been nothing but small efforts at research, even if a few have been well-meaning and well-conceived. NIH allocates only 15 million dollars per year for ME/CFS research but, just a few years ago, it was only 6 million dollars per year [25]. Multiple Sclerosis is thought to be, on average, much less severe in its impact on patients’ quality of life, and affects half the number of people (at least, the number of affected MS patients is likely accurate, the number of estimated ME/CFS patients is likely very inaccurate). However, MS receives 100 million dollars per year from the government for research. HIV receives 28 billion dollars per year [25].
- Some patients are committed to psych wards [26]. This probably happened a lot more in the past. The number is thankfully declining, but it still happens. There is one woman who was forced into a psych ward and, while there, the clinicians, at one point, threw her into a swimming pool to try to force her to “take initiative”, or something. She almost drowned. She got much, much worse while kept at the psych ward but did finally get out after relentless help from the ME/CFS community or family/friends (I’m not sure which) [26]. I’m sure there are many diagnosed and even more undiagnosed ME/CFS patients around the world being forced into treatments and forced to take medications that harm them, getting worse and worse and suffering profoundly as a direct result of being locked in psych wards. I recently wrote a letter to a hospital that is currently threatening to lock up an ME/CFS patient in Sweden against his will. It is included in the appendix below (see Appendix A.6).

### Appendix A.2. Staying below Energy Limits

My number one piece of health advice for Chronic Fatigue Syndrome/Myalgic Encephalomyelitis patients, more important than any current medication or treatment, is to never exceed your energy limit. Let me explain to be clear. The most unique, best identifying symptom of Chronic Fatigue Syndrome/Myalgic Encephalomyelitis (ME/CFS) is Post-Exertional Malaise. Healthy people can exercise way past the point of exhaustion. They can continue when their bodies scream at them to stop and later that day, they recover and feel fine, if not euphoric. I know this feeling well; I ran cross-country in high school. ME/CFS patients have an energy limit and if we exceed that limit, we get Post-Exertional Malaise, which means we get physically sick afterward, and any ME/CFS symptoms we have get worse. This can last for days, weeks, months or be a permanent worsening of the illness. The most important part is that, when we exceed our energy limit, the limit goes down, so next time we have to stop sooner or the whole process repeats itself as a vicious cycle.

Patients with mild ME/CFS usually only reach their limit with anaerobic exercise, but a more severe patient’s limit can be brushing their teeth for too long a time and, for even worse patients like me, the limit is, for example, being touched by another person, being in the same room with someone else, looking at something for too long or even thinking about something for too long, or thinking of something that requires too much mental energy. Having someone in the room, especially, puts me over my limit. The combination of thinking at the same time is extremely overwhelming. I have to meditate on a couple simple ideas or memories, and if my mind strays, even for a moment, it can be devastating.

Most people are completely out of touch with their bodies. ME/CFS patients have to learn to be keenly aware of our bodies and exactly where our limit is. We have to make a choice to stop when we feel ourselves reaching our limit before we go above it. This choice is part of the reason we are judged so harshly by friends, family and even loved ones: we have to choose to stop activities or refrain from activities before they make us symptomatic. People don’t see us getting Post-Exertional Malaise—we go home and collapse, suffer, sleep or rest in private. Due to this lack of awareness, they never understand the connection.

It’s also extremely difficult for us because it’s pleasure first, negative consequences later, and our minds are famously bad at negotiating this. Think of how hard it is to stop using drugs. It’s a similar pleasure first, negative consequences later situation. With ME/CFS, it is life itself we are having to refrain from, not a high from life.

A good way to control the urge to indulge the pleasure center of the mind is to think about how you will feel afterward. If you exceed your energy limit pushing yourself to continue engaging in an activity—mental or physical—your limit will go down, you may never be able to do that activity again, and, in the future, you’ll have to do even less just to stay below your new limit. It helps to bring the negative consequences into the present and hopefully make it easier for you to stop within your limits.

This happens to be Ronald Davis number one piece of health advice as well, arrived at independently of me. Genius minds think alike.

Please understand, getting worse DOES NOT mean this is your fault. For one thing, ME/CFS is an extremely complicated illness, and its mechanisms are mostly unexplained to date. Some people (like me) get worse without exceeding their energy limits. I went from mild to severe ME/CFS overnight while traveling in India. Then, I slowly recovered and went back to ME/CFS severe over and over until I came home and got better, but remaining worse than before India. No one knows what caused this. The world is also full of chaos, and we can’t always accurately predict how much energy something will take. We often get stuck in situations we can’t just stop, and we have to push ourselves past our limits. This is why a predictable routine becomes important—the less unexpected energy expenditure, the less likely we are to wind up in one of these situations and overdo it. It’s also really hard to know your limit and body well enough to feel it coming on and stop. It takes years of experience.

So don’t blame yourself. Just do your best and let go of the rest, and prepare for the very real likelihood that you could get worse.

### Appendix A.3. The True Horror Of ME/CFS

From the CDC, “According to an Institute of Medicine (IOM) report published in 2015, an estimated 836,000 to 2.5 million Americans suffer from ME/CFS, but most of them have not been diagnosed.” [27].

This number is generally stated as being about 2 million, the higher end of the CDC’s estimate [28]. However, I’m not sure how the CDC or anyone else thinks they can accurately guess at the number of people afflicted by an illness for which it is so difficult to simply obtain a diagnosis. What logic or thought process lead them to the ”2 million” number?

In honor of all those who lie in silence and darkness, to those whose terrible deaths which were marked as cause “unknown” or “heart attack”, etc., and to those who have taken their lives due to unbearable suffering, I must relay to you the unfortunate true horror of ME/CFS. It is much worse than estimates like this and has been for at least forty years. I know this by looking at what we know and using simple logic to extrapolate from there. These are the logical steps:

(1) We only hear from people who are diagnosed. How hard was it for you to get diagnosed? It took me about 7 years of constantly seeing doctors. I was told my symptoms would resolve themselves, or that nothing was actually wrong with me, or there was just no answer and nothing else to test for, or the famous “it was in my head and not a physical illness”. How many people have the fortitude to keep going in the face of this, and for how long? It takes a very specific kind of person to be utterly relentless enough to continue pushing doctors to dig deeper and continue seeing new doctors for second, third, fourth, fifth—and even more—opinions, all the while ignoring the blatant prejudice and disrespect constantly shown to them. Everything pushes ME/CFS patients to give up and try to either live with their “health problems” (what I called it for years) or kill themselves. Everything. What percentage of regular people out there who have ME/CFS have the chutzpah to keep fighting for answers in spite of all of that? 10%? 5%? Less? [29].

(2) Of that small percentage of ME/CFS patients who are diagnosed, how many are lucky enough to remain mildly sick and not get worse, despite holding a job, taking care of kids, feeding themselves, feeding a family and doing all the myriad other things it takes to be an independent adult?

(3) Of those who get diagnosed but become too sick to care for themselves, how many are lucky enough to have family or loved ones who will support them and take care of them? It’s worth noting that it’s much less likely that undiagnosed ME/CFS patients will have family/friends/loved ones who will be understanding enough to help them or take care of them. Even for diagnosed patients, there are few people in the world who have access to the care that is necessary to keep someone who is severely ill alive. I know how lucky I am. It takes loved ones who are willing to give up their personal and professional lives, with enough money to pay for huge medical bills that aren’t covered by insurance, because we don’t have a “legitimate illness”.

(4) How many are willing or able to carry on emotionally, continuing to survive the horrifying “living death” that defines severe ME/CFS?

(5) Of those who manage to get (1) a diagnosis and (2) are lucky enough to either not get worse (rare, especially if they are trying to maintain a healthy person’s workload, which is very common) or (3) have people in their lives willing and able to take care of them and (4) are emotionally able to carry on despite the incredible suffering ME/CFS inflicts, how many of this dwindling percentage are interested in social media/find the forums and social media pages, and then how many are even capable of using computers (many ME/CFS patients are not able to)?

This is what it takes to be “seen” as an ME/CFS patient. The people on Facebook, Twitter, Phoenix Rising and other ME/CFS forums are the very tip of the iceberg. We only hear from people who make it through these five tiers. It’s likely that this is a very, very small percentage of ME/CFS patients [29].

What happens to the rest of the diagnosed/undiagnosed ME/CFS patients in the country/world? This is something no one talks about. If you just look at the facts, human nature and how our society functions, it suddenly becomes horrifying. They must wind up on the streets, getting worse and worse until they die a terrible death alone in a gutter somewhere [30]. I believe this happens to a huge number of ME/CFS patients.

How many people are as sick as I am? Only a few of us are publicly known, but surely there are staggering numbers of people as sick as I am. (Even if the estimates are correct and one quarter of the “2 million” is severely ill, that’s at least 500,000, just in the U.S.)

I’ll ask again: what happens to all the rest of us? My fellow severe ME/CFS patients have either killed themselves or will die alone in a ditch; likely hundreds of thousands of us or more. In a ditch. Alone. We need the support of the federal health agencies to fund research and care programs (ME/CFS wards that house ME/CFS patients who don’t have anyone to take care of them, and can cater to their sensitivities) for this disease, and, yet it hasn’t been offered. So many of us have just been left to die alone.

### Appendix A.4. Adjusting Expectations

I think one important aspect of coping with ME/CFS is lowering our expectations, as sad as that is to do. A discrepancy between expectations and reality is one of the biggest causes of unhappiness, even among healthy people. If your happiness depends on something you don’t have, you will be unhappy. Living with ME/CFS is a process of lowering the bar of expectations you once had for your life. You have to do it, or you’ll go crazy. Since ME/CFS is often degenerative, as it was for me, it becomes a process of continuing to let go of expectations and continuing to lower the bar until, as in my case, it’s practically on the ground. There was a time when I said I would kill myself if I ever had to move in with my parents. Yet here I am alive, after living with them for about 10 years now, since 2011.

One way to do this is to try to be open minded to things you once thought were beneath you, or simply not befitting your personality or the way you wanted to live. I did a lot of this.

When I became housebound while living in Berkeley, California, I realized how difficult it was going to be to meet people while stuck in my house, so I signed up for a dating website, which is something I never would have done before I was sick. I decided I needed to be open minded to the options that were actually on the table to maximize my quality of life. Nothing much came of the dating website, except one really awesome girl, who made it all worthwhile. We had a good, short relationship—a few months—before my illness got in the way and the relationship ran its course, and we ended things on good terms.

I also really worked on overcoming shyness and asking girls out for coffee/tea who I didn’t know but met randomly in public. I met a girl this way too—an employee at the Whole Foods I went to for groceries. (One of my few outings—which exhausted me.)

Being creative is hardwired in my existence and the worst parts of this illness have been when I’m too sick to be creative with anything in my life. As long as my health allowed, I’ve always tried to find creative projects I could handle working on within my energy limits. One thing I did in Berkeley, and, while my health permitted, here at my parents’ house, was collect headphones with good drivers that had poor acoustic implementation, resulting in much inferior sound than they were capable of. I learned how to add acoustic implementation that allowed the drivers to operate better so the headphones would sound as good as they could, resulting in some incredible-sounding headphones. I had to give up the kind of photographs I used to make, but things like this somewhat filled the creative void that was left in my life.

For the last 7 years, I haven’t had the energy or freedom in my daily routine to be creative at all, and it’s been crushing. I’ve felt adrift and empty. However, I hung on for the ride and now I’ve had a completely unexpected upswing, and I get to try to be creative with these social media pages and writing. Again, it’s not what my healthy self would be doing but I have to change my expectations and adjust and be open-minded, and then I can find happiness in things I wouldn’t have before.

I even love my iPhone now, which is something that would have been an anathema to me before. It’s now my only connection to the world and only way of engaging with the world. When I get better, hopefully it won’t remain attached to my hip, but if I’m better it won’t matter—I’ll be better!

### Appendix A.5. My Whole World Exists in Bed

Something I don’t think people who haven’t experienced being bedridden understand is that, when you’re bedridden, your whole world exists in bed. You don’t climb into bed to sleep or nap or get cozy and then get out of bed and live in the rest of the room/house/world.

You are always in bed. It’s your whole world. I think this contributes to some of the sensitivity that severe ME/CFS patients experience. Anyone would be particular about their bed if they were bedridden, but it is also exacerbated by the sensitivity that the illness causes.

Half of my bed is dedicated to me and the other half to storing things I need access to because I can’t get up to get things. On my bed are: my stomach massagers (for my severe gastroparesis), ice for my stomach (ice helps my stomach feel and function better), a stack of paper towels, remote door bells which I use as call buttons when I need something, a container of water for cleaning or rinsing off baby wipe soap, a basket with odds and ends like the remote control to my A/C, masks for the smoke from the forest fires before I got an air purifier, the towel I use to cover my eyes when people come in, my white noise earphones, my earmuffs, a stack of adult diapers because I got a urine infection once and had trouble holding it in time to get a urinal and now I keep them accessible just in case, a little jar for trash, boogers, etc.—you get the idea. I’ve packed as much as possible within reach under my bed, like a vibrating massager for my feet and legs, which get restless leg syndrome (tingly feeling in the legs and feet that can be unbearable) from being so still, or nervous system weirdness—I don’t know which. I also store extra backup stomach massagers in case one fails, my heating pad, which I use on my feet to keep warm since I have ice on my stomach all the time, and lots more.

All these items are, to me, like all the stuff in your house. You are just as particular about how your house is arranged as I am about how my bed is arranged, and you get to leave the house and get away from all that stuff and move freely with a few possessions. I don’t. This bed is where I reside 24/7 and I need access to this stuff 24/7.

Some symptoms of severe ME/CFS are partly just normal reactions to horrid nightmare living conditions. They are, of course, compounded by the sensitivity that severe ME/CFS causes. However, I think it’s important for caregivers, doctors and healthcare professionals to understand how challenging living conditions are with severe ME/CFS, and the fact that any healthy person would also react adversely and wind up acting “abnormally” in response to these conditions. It is, in fact, not an abnormal reaction, nor is it a sign of mental or psychological illness. It is a healthy, pro-active response to the limitations imposed by the illness, making it easier to access things with minimal energy expenditure. For example, I have a piece of tape on the floor marking where my bed urinals should be precisely lined up. If they are always in the same place, I can develop muscle memory for the action of reaching down and picking them up and can do it with very little thought or energy. I can even reach them with my eyes closed. I try to have everything in my bed like this. Again, it is not OCD.

### Appendix A.6. A Hospital in Sweden Is Threatening to Commit a Severe ME/CFS Patient to a Psych Ward

Holger Klintenberg is a severe ME/CFS patient in Sweden who is being threatened by a local hospital with committing him by force to their psych ward. He is extremely severe and this will kill him.

This is a letter I wrote to the Hospital that is threatening to commit Holger.

Dear *LänssjukhusetRyhov* hospital threatening to commit Holger Klintenberg against his will,

My name is Whitney Dafoe and like Holger I also have severe ME/CFS. I have recently seen some minor improvement from an experimental drug that reduces brain inflammation which is the only reason I’m able to write this now. I spent four long years in a state very similar to the condition Holger is in.

I’m writing you to tell you that if I was committed to a psych ward even now it would without question kill me. It would have killed me faster if I was committed when I was in Holger’s condition. Holger will die if you commit him to a psych ward. Period. If you doubt this you should watch this news clip about an ME/CFS patient who died as a direct result of being committed. And ask yourself: do you want that on your conscience? Do you want that kind of publicity? Killing someone? Because you will get it.

https://youtu.be/yrBAlKtroBw (accessed on 22 April 2021)

ME/CFS is not a psychological illness. It never has been and there has never been an acceptable reason to treat ME/CFS patients the way you are threatening to treat Holger. For decades there has been so little research into ME/CFS that there hasn’t been a lot of proof of physical illness. However in recent years there has been a surge of research into ME/CFS due to a team of world renowned scientists (all award winning and 3 Nobel prize award winners) at Stanford University taking on the illness full steam, almost entirely privately funded. They have made a number of profound discoveries in only a few years that prove this is a real physical illness and they are only going to find more proof as they move closer to finding a diagnostic test and a cure. The lead researcher is Ronald Davis and he would be happy to speak with you about these discoveries and give you his informed opinion on the consequences of committing Holger. How will you feel after killing someone who could have lived to see a cure discovered and experience a full recovery?

The main, most distinguishing symptom of ME/CFS is something called Post Exertional Malaise (PEM) which refers to symptoms worsening with physical exertion, or with severe patients like Holger and myself, mental exertion as well. Patients with ME/CFS have what is called an energy envelope—in other words—an energy limit. When patients exceed this limit, two things happen. Their symptoms get worse which can last for hours, days, months, or years. And most importantly, the energy limit lowers. I got worse for 4 years due to going over my energy limit for one too many days.

Holger is in an extremely fragile state. Because he is so severe, his energy limit is so low that even the most mild stimulation such as light or noise forces his mind to use more energy than his limit permits and he gets worse. And his energy limit goes down even further. This is an extremely dangerous vicious cycle where every time the energy limit is exceeded, it gets lower and the patient has to figure out how to live, or in Holger’s case, survive, while staying under this limit. At some point, if he is not in an environment that allows him to do this, the limit will get so low that he will not be able to stay under it and he will quickly get worse at an exponential rate until he dies.

This is not an idea or theory. This is the reality that millions of people suffering from ME/CFS face every day among other devastating symptoms.

If you commit Holger to a psych ward, it will kill him without question. If you do not commit him and allow him to live in the space he has been living in, he will very likely survive long enough for the research team at Stanford to find treatments that will make him much better or a cure that will return him to a fully healthy, productive member of society.

You have a choice to make and you now know what the consequences of that choice will be. If you have any semblance of humanity or decency you will give Holger a chance to live. That’s the least that any human being deserves.

Thank you for your time and consideration.

Sincerely,

Whitney Dafoe, severe ME/CFS patient

www.whitneydafoe.com/mecfs (accessed on 22 April 2021)

www.facebook.com/whitneydafoe (accessed on 22 April 2021)

www.twitter.com/dafoewhitney (accessed on 22 April 2021)

www.instagram.com/whitneydafoe (accessed on 22 April 2021)

### Appendix A.7. Good Science Grants Being Turned Down by NIH

Ronald Davis and other good ME/CFS scientists’ brilliant grants are currently being turned down by NIH. Part of the reason for this is that the system for grant review is a mess [31]. Grants are reviewed by study sections, whose reviewers give them a score. They then go to Council, which funds the ones with the best scores. The main problems are: (1) There is only money for about 10% to get funded. (2) Reviewers nit-pick the grants so only a few get good scores, when, in fact, a much larger percentage of the grants are good. (3) Reviewers are often underqualified and uninformed about the subject of the grant, so their criticisms are incorrect—at times, ridiculous. (4) Council takes these inaccurate reviews as gospel and just funds the top few, without any evaluation of the competence of the reviews or consideration about the importance/urgency of the science. (5) *The leadership at NIH is obviously not committed to addressing the urgent problem of millions of people suffering from the horrific disease of ME/CFS.*

What is required is that scientists focus on researching things that can actually make a difference and lead to treatments or a cure for this disease. Grant reviewers are looking for a hypothesis that can be researched and lead to an answer that can be published. Ronald Davis isn’t thinking about getting published; he’s trying to find answers to what’s happening inside the bodies of ME/CFS patients and discover an intervention that might help. What he wants to do isn’t always so simple as “hypothesis, research, publication” and the grant reviewers only like grants that use existing, well-established methods that will lead to a publication. They can’t imagine that anything they have never seen before could actually work. All of Dr. Davis’ grants involve things they have never seen before. They don’t understand it, which, when combined with what a mess ME/CFS is, makes it even harder for them. A lot of them also probably know nothing about ME/CFS or are prejudiced against it to begin with.

Another thing that gets in our way is, actually, probably a good thing most of the time. NIH has a rule that they are not allowed to communicate with the grant reviewers. I believe it’s to try to keep things impartial. However, this rule hinders NIH from intervening and urging acceptance of Dr. Davis’ grants and other good ME/CFS grants to try to make it impartial as it should be. I don’t believe NIH is allowed to pick who reviews which grants, either. However, the Council’s JOB is to make certain the reviews are competent and unbiased, and that the research addresses urgent and nationally important topics. In the case of ME/CFS, their job should be to make sure ME/CFS has adequate funding and to make certain that the research is likely to make progress towards understanding the disease in a way that might lead to treatment and cure. Not just a bunch of random data to publish.

This is all true, but to offer this as the cause of the problem presumes that the various heads of NIH actually want these grants approved in the first place.

What is also true is that NIH is engaged in a duplicitous publicity stunt, trying to continue their 40-year campaign of intentionally ignoring ME/CFS and systematically denying grants simply because they relate to ME/CFS, while, at the same time, trying to cultivate a public image of supporting ME/CFS. NIH has recently been saying things like “we want to and are ready to fund ME/CFS grants ’based on good science’ so turn in grants and we’ll fund them”. Sounds good, right?

However, when good science grants about ME/CFS are submitted to NIH, these scientists review them and find absurd reasons to give them bad scores so they then get dismissed as “bad science”.

I’ve got news for you, Francis Collins (the Director of NIH). Ronald Davis doesn’t write, speak or think “bad science”. We see the game you’re playing, and we think you are an even more depraved human being for playing it. Either do the right thing and fund worthwhile ME/CFS grants, or publicly face the consequences of the blatant prejudice you are enacting.

We know your system is difficult, but we also know that you are the Director and you are capable of intervening when there is a severe health crisis, so that it gets addressed. It’s been done before. You just have to believe that we have a real disease, that we are suffering, that more people will suffer, and that science needs significant funding to end the disease and end the suffering. You have to care. You told us “We are the National Institutes of Hope”, “We are a family, in this together” and “We are ready to fund good grants”. You need to put your money where your mouth is. You know what a good grant looks like.

This isn’t something you’re going to get away with. We all see what you are doing, will remember it, and history will record it.

### Appendix A.8. My Experience in a Wheelchair

Have you ever had to use a wheelchair because of ME/CFS? I have and found it to be an unexpected experience. My legs slowly got worse because of circulation problems (I think) to the point where I could only walk into the kitchen once per day (15 feet or so). Then it got worse, and I had to crawl. I couldn’t get a wheelchair from my insurance company because, even though I couldn’t walk, I had no diagnosis they considered valid. When I asked my primary doctor (who I’d been seeing for years, trying to get a diagnosis, before I got diagnosed) for help, he said he thought it would be bad for me and he couldn’t in good conscience help me get a wheelchair (because he thought getting me a wheelchair would reinforce my “non-existent illness that was in my head” and that I “needed to get over”). I finally got one on Craigslist for cheap. At first, I just used it to get around the house, which was a huge help. No more crawling to the kitchen for ice cream. It also gave me more independence to microwave my own food from leftovers. The real surprise came when I went out in public with it—mostly to Drs. appointments.

The way people treated me was a revelation. They instantly knew there was something dysfunctional in my body and treated me with respect, let me go first, and kind of bowed in respect to the hardship I was facing. It wasn’t that I was craving attention, but after the way my friends and doctors treated me and the lack of funding and support from society, it was a shocking polar opposite that honestly felt really, really good. It was amazing to feel instant recognition from people of a real illness.

Most people don’t feel good about being seen in public in a wheelchair, so this illuminates just how badly I was treated, and how many people in my life were constantly questioning the validity of my illness. When seen in a wheelchair, there was no question, just instant recognition and understanding.

### Appendix A.9. When Life Gives You Rotten Lemons

They say, “When life gives you lemons, you make lemonade”. What do you do when life gives you rotten lemons?

First, you are overwhelmed with anger that you didn’t even get fresh lemons. In time, the anger turns to sadness, and slowly you start longing for fresh lemons.

You spend all your available energy thinking about making lemonade. How you would squeeze them, all the ingredients you could use. You become the most incredible lemonade maker in the world, only you’re stuck at home, or in bed.

I can only imagine the torrent of knowledge and wisdom that will be unleashed upon the world when we are all cured.
